# Assessing Problematic Social Media Use in Adolescents by Parental Ratings: Development and Validation of the Social Media Disorder Scale for Parents (SMDS-P)

**DOI:** 10.3390/jcm10040617

**Published:** 2021-02-06

**Authors:** Maria Isabella Austermann, Rainer Thomasius, Kerstin Paschke

**Affiliations:** German Center for Addiction Research in Childhood and Adolescence (DZSKJ), University Medical Center Hamburg-Eppendorf (UKE), Martinistrasse 52, D-20246 Hamburg, Germany; ma.austermann@uke.de (M.I.A.); thomasius@uke.de (R.T.)

**Keywords:** social media disorder, adolescents, parental rating, Internet-related disorder, questionnaire

## Abstract

Background: The problematic use of social media (SM) is a rising phenomenon, especially in adolescents. It can be assessed by self-rating screeners such as the Social Media Disorder Scale (SMDS). However, young age or symptom denial might reduce adolescent assessment accuracy. Therefore, the development and validation of a parental scale (SMDS-P) is desirable. Method: A representative sample of 961 parents and corresponding frequently SM-using children aged 10 to 17 years participated in an online study. Factorial analyses were performed to determine item structure. Adolescents’ SMDS self-reports, SM usage time, emotional dysregulation, and academic performance were used to assess validity. The SMDS-P cut-off value was calculated by ROC-analysis. Results: A one-factorial structure of the SMDS-P could be confirmed. The internal consistency was good (Cronbach’s α = 0.85, McDonald’s ω = 0.88) and the accordance between parental and self-ratings moderate (kappa = 0.51). SMDS-P was positively associated with adolescents’ self-ratings (r = 0.68), SM usage time (r = 0.26) and frequency (ϱ = 0.16) as well as with emotional dysregulation (r = 0.35) in a highly significant manner. Conclusions: SMDS-P offers a promising new approach to assess problematic SM usage in adolescence. Further studies including clinical validations are required.

## 1. Introduction

With the growing popularity of social media (SM) in our society, the number of adolescents using them regularly has significantly increased, especially during the current COVID-19 pandemic [1]. In addition to their advantage of connecting people, SM services also entail an addictive potential due to psychological mechanisms built in by developers to achieve strong user loyalty and high usage times [2]. Since puberty is associated with both peaks in the desire to experience sensations, novelties, and rewards as well as limitations in the capability to exert cognitive control [3], adolescents are considered particularly at risk for the development of problematic SM use (PSMU). PSMU resembles pathological and at-risk addictive behavioral patterns and is often accompanied by symptoms of mental disorders such as depression, anxiety disorders, attention deficit hyperactivity disorder, obsessive compulsive disorder, and disordered eating [4,5,6]. Affected adolescents show problems in their emotion regulation [7] which is considered a core component of psychiatric diseases [8]. Moreover, PSMU, as well as increased SM usage times in general are associated with academic performance deterioration in adolescents [9,10,11]. Significant correlations between the frequency and duration of SM use and PSMU could be repeatedly shown [9,12,13,14].

Albeit suggested [15], PSMU has not yet been included in diagnostic manuals. Scales to assess PSMU in adolescence or young adults are often based on the general criteria of addictive disorders (cf. Six Core Components of Addiction Model by Griffith) [16,17,18,19] or on the related construct of Internet Gaming Disorder (IGD) [12]. The latter has been included as a condition warranting more research in the fifth edition of the Diagnostic and Statistical Manual of Mental Disorders (DSM-5) as the first and to date only member of Internet-related disorders [20]. For an IGD diagnosis, five of the following nine criteria must have been met for at least 12 months: preoccupation, withdrawal (when not using), tolerance, unsuccessful attempts to reduce or stop usage (persistence), continuation of usage despite problems (problems), deceiving or covering up usage (deception), usage to escape adverse moods (escape), giving up other activities (displacement), and risking or losing relationships or career opportunities due to excessive usage (conflict). 

The Social Media Disorder Scale (SMDS) by van den Eijnden et al. (2016) [12] is oriented towards the IGD criteria and represents a short and easy to use self-rating questionnaire to assess PMSU. It was translated into different languages, validated in samples of adolescents in Europe and China and proved to be a psychometrically sound instrument [12,21,22]. It was recently applied to estimate the prevalence rates of PSMU among 10- to 17-year-old adolescents from 29 European countries [23]. The values ranged from 3.22% (Netherlands) via 5.35% (Germany) to 14.17% (Spain), with an average prevalence of 7.38%. These numbers underline the importance of this issue.

It has to be noted that the currently available screening tools for PSMU including the SMDS have been solely based on self-reports. Although the use of such scales in later childhood and adolescence has repeatedly considered valuable [24], the ability to reflect on thoughts and behaviors is not fully developed until late adolescence [25] potentially reducing validity. Moreover, self-regulation and executive control functions are less pronounced in individuals with the problematic use of Internet services in general and linked to poor introspection [25,26,27,28]. Self-reports on addictive behaviors are often confounded by socially desirable response patterns, symptom denial and concealment or exaggeration [29,30,31]. A comparison of self-ratings on IGD and clinical interviews by trained mental health professionals showed a false negative rate for the self-reported IGD assessment of 44% and a false positive rate of 9.6% [32]. Self-report biases can be controlled by having a professional assist the respondents in answering the questionnaire or by using additional items that measure, for example, the tendency to give socially desirable answers [33]. Another promising approach is the assessment of adolescent PSMU by external ratings. For this, the involvement of the parents of the affected adolescents might be particularly suitable.

Parents are often the first to notice problems in their children’s behavior. In addition, parents often initiate contact with the clinician or accompany their children to the medical consultation, so they can easily be included in a screening or diagnostic process. In contrast to PSMU, parental rating scales for the assessment of problematic gaming [34,35] and Internet use [36,37] among adolescents already exist. The parental version of the Internet Gaming Disorder Scale (P-IGDS) is like the SMDS oriented to the DSM-5 criteria for IGD. The scale showed high internal consistency and promising criterion validity [34]. It is to be examined whether adapted IGD criteria prove their validity in the context of a parental assessment on adolescents’ problematic SM usage behavior as well.

Given the prevalence of PSMU, the rising SM usage times, and the limitations of self-rating scales, a validated screening instrument to extend the assessment of PSMU in children and adolescents by parental ratings is urgently needed but, to the best of our knowledge, not available at this point. Therefore, the present study aimed (1) to adapt the SMDS as a parental version; (2) to investigate the psychometric properties of the new scale; (3) to validate it in a representative sample of parents and their 10- to 17-year-old frequently SM-using children; and (4) to determine the accordance between parental and adolescent ratings. 

## 2. Methods

### 2.1. Participants and Procedure

The data were collected by an established German market and opinion research company as part of an online survey on media usage in German families. For a detailed description of the study design and the sample recruitment, please refer to Paschke et al. (2020, 2021) [38,39]. A total of 23,736 German households with adults aged from 28 to 75 years were contacted by e-mail in the period from 13 to 27 September 2019. These belonged to a large representative sample of German citizens. A total of 12,427 adults responded to the e-mail. Among these, 1733 indicated that they had children between 10 and 17 years of age. Of these, 1221 parents and one corresponding child each gave their consent to participate in the survey and provided the necessary information. In households with more than one child aged 10–17, the child with the most recent birthday was asked to participate in the survey. Representativeness was ensured in terms of gender, age, and residential region. The estimated time needed to complete the survey was 20 min. The survey was divided into two parts. The first part was addressed to the parents, and the second to the adolescents. Adolescents were asked to answer the questions as independently as possible, but they could consult their parents on comprehension questions. The parents were asked to not suggest any answers. Participants could withdraw from the study at any time for any reason.

A total of 1055 parents reported their child’s frequent SM use (at least once a week). Their data and those of their children were included in the further analysis (N = 2110 in total).

The study was conducted in accordance with the relevant national and institutional committees on human experimentation, in compliance with the Declaration of Helsinki, and was approved by the Local Psychological Ethics Commission at the Center for Psychosocial Medicine (LPEK) of the University Medical Center Hamburg-Eppendorf (UKE).

### 2.2. Measures

#### 2.2.1. Problematic Social Media Use (PSMU)

The Social Media Disorder Scale for Parents (SMDS-P) was used in this study to obtain an external assessment of PSMU among the participating adolescents. The SMDS-P was developed based on the SMDS by van den Eijnden et al. (2016) [12]. For the SMDS-P, the 9 statements of the SMDS were adapted to address the parent’s opinion on their child’s behavior. The content and the 12-month-time criterion of the scale were retained as well as its binary response format. Table 1 lists the items of the SMDS-P and the corresponding DSM-5 IGD criteria. The German version of the SMDS-P is presented in Table S1 (see Supplement). Analogue to the SMDS, the response options of the SMDS-P were coded with “0” for “no” and “1” for “yes”, so that a maximum sum score of 9 could be achieved. 

To determine whether the SMDS-P was consistent with the adolescent’s self-reports on PSMU symptoms, the adolescents were asked to fill out the SMDS. In accordance with the DSM-5 recommendation for the diagnosis of IGD, a sum score higher than 4 was considered as an indication for problematic usage behavior [12]. The internal consistency of the SMDS for the sample of the current study was good (Cronbach’s α = 0.84) [40].

#### 2.2.2. Adolescent Usage Time, Emotional Dysregulation, and Academic Functioning

In this study, SM were defined as all digital services on which texts, photos, animations, or videos can be shared, commented on or liked (e.g., Instagram, TikTok, YouTube). The weekly SM usage frequency of the participating adolescents (measured by the average number of usage days per week) as well as their average usage time on days during the week (resp. school or working days) and at the weekend (resp. non-school or non-working days) was estimated by both the adolescents and their parents. Based on the reported usage times, an average daily usage time was calculated.

Difficulties in emotion regulation were measured using the homonymous scale by Kaufman et al. (2016) [41]. The short form of the Difficulties in Emotion Regulation Scale (DERS-SF) comprises 18 items. An example item of the DERS-SF is: “When I’m upset, I become out of control”. Answers can be given on a 5-point Likert scale, ranging from 1 (“almost never”) to 5 (“almost always”) for negatively and inversed for positively phrased items. A maximum sum score of 90 is possible. The scale is scored so that higher values reflect greater difficulties in emotion regulation. Cronbach’s α of the DERS-SF was 0.90 suggesting excellent internal consistency in our sample.

Adolescents’ school/work absenteeism was surveyed in days for the last three months. School performance was assessed based on the last reported grades in the three main subjects (German, mathematics, first foreign language), which ranged from 1 (very good performance) to 6 (unsatisfactory performance). The three grades were combined for a grade sum score, with higher values indicating poorer school performance. Furthermore, the development of the grade sum score in the past school year was examined by asking the adolescents to compare the current grade sum score with the grade sum score of the last school report. The degree of change in the grade sum score was coded as follows: 1 (significant decline), 2 (mild decline), 3 (constant performance), 4 (mild improvement) and 5 (significant improvement). Thus, higher numbers indicated greater improvement.

### 2.3. Statistical Analysis

#### 2.3.1. Data Cleansing

Data from the subjects with missing values of more than one third for each instrument (SMDS-P, SMDS, and DERS) were excluded from further analysis (N = 94), leading to a final sample size of N = 961 parent–child dyads (N = 1922 participants in total). Missing values were estimated by multiple imputations, using the package mice of the statistical program R [42,43]. This resulted in total replacements per instrument of 3.65% (SMDS-P), 1.55% (SMDS) and 1.93% (DERS-SF). 

#### 2.3.2. Factor Structure

The sample was randomly divided in two (nearly) equal proportions by a split-half validation method using the R package rsample (n1 = 481 dyads; n2 = 480 dyads) [44]. Afterwards, an exploratory factor analysis (EFA) was performed on the SMDS-P items for the first half of the sample. Then, a confirmatory factor analysis (CFA) was conducted for the second half to test for the replication of this factor structure using the R packages psych and lavaan [45,46]. The Kaiser–Meyer–Olkin (KMO) criterion and Bartlett’s test of sphericity were determined to assess the data for the suitability for factor analysis. For the revision of normality distribution, absolute values of skewness >2.0 and kurtosis >7.0 were used as reference values to determine substantial non-normality [47]. Since SMDS-P includes ordered categorical variables, a robust minimal residuals (OLS) factoring was conducted [48]. For the first subsample, the Wayne Velicer’s Minimum Average Partial (MAP) criterion was used to determine the appropriate number of factors. The goodness of fit of the component structure tested with CFA was evaluated by the χ^2^/df ratio (<5), the root mean square error of approximation (RMSEA < 0.08), the standardized root mean squared residual (SRMR < 0.08), the Tucker–Lewis Index (TLI ≥ 0.95), and the comparative fit index (CFI ≥ 0.95) [49].

#### 2.3.3. Internal Consistency

Internal consistency was computed by Cronbach’s α and McDonald’s ω. Both coefficients were interpreted as follows: ≥0.9—excellent; ≥0.8—good; ≥0.7—acceptable; ≥0.6—questionable; ≥0.5—poor; and <0.5—unacceptable [40,50].

#### 2.3.4. Criterion Validity

Criterion validity was determined by the correlation of the SMDS-P sum score with the SMDS sum score, the weekly SM usage frequency, the mean SM usage time per day (as reported by the adolescents), the DERS-SF sum score, days of school/work absenteeism, the grade sum score, and grade development applying Pearson or Spearman rank correlation tests (depending on the item/scale distribution). Absolute correlation coefficients were interpreted as follows: Pearson: 0 ≤ r ≤ 0.10 zero or negligible relationship; 0.10 < r ≤ 0.30 weak relationship; 0.30 < r ≤ 0.50 moderate relationship; r > 0.5 strong relationship [51]; Spearman: 0 ≤ ϱ ≤ 0.10 zero or negligible relationship; 0.1 < ϱ ≤ 0.40 weak relationship; 0.40 < ϱ ≤ 0.70 moderate relationship; 0.70 < ϱ ≤ 0.90 strong relationship; ϱ > 0.90 perfect relationship [52]. 

#### 2.3.5. Sensitivity and Specificity 

A receiver operating characteristic (ROC) curve analysis was conducted to compare sensitivity and specificity across SMDS-P scores to predict PSMU according to the SMDS classification [53]. Moreover, 999 bootstrapping replications were applied for the definition of 95% confidence intervals (CI). Cut-off points were determined on the basis of Youden’s criterion, considering sensitivity and specificity to an equal extent. Goodness of differentiation between the two diagnostic groups was computed by the area under curve (AUC) value [54]. The received cut-off point was then applied to classify adolescents with problematic usage patterns. Groups were compared regarding age, sex, SMDS-P and SMDS score, SM usage days per week, mean SM usage hours per day, school/work absence, grade sum score, and grades development by a MANOVA with post hoc χ^2^ and Scheffé tests. Effect sizes to compare the problematic and normal usage groups were computed using Cramer’s V (categorial variables) and Cohen’s d with the following interpretation of the absolute values: Cramer’s V > 0.5 strong, >0.3 moderate, >0.1 weak effect [55]; Cohen’s d > 0.8 large, >0.5 medium, >0.2 small effect [56].

#### 2.3.6. Accordance Rate

The accordance rate was determined by comparing the SMD classification based on SMDS with the one based on SMDS-P using Cohen’s kappa (unweighted). Cohen’s kappa values were interpreted as follows: >0.8 almost perfect accordance, >0.6 substantial accordance, >0.4 moderate accordance, >0.2 fair accordance [57]. 

## 3. Results

### 3.1. Sample Description

Of the 961 parents who reported a frequent SM use of their child, 54.0% of them were mothers (n = 519) and 46.0% fathers (n = 442). Their mean age was 46.40 years (standard deviation (SD) = 7.87, range = 28–75). Nearly one-third (28.2%) of the parents had a high educational status (n = 271); 60.8% (n = 584) had a medium; and 8.8% (n = 85) a low educational status. However, 2.2% (n = 21) of the parents could not be assigned to a status based on their answers regarding their education. Among all the parents, 59.1% reported working full-time (n = 567) and 29.6% worked part-time (n = 284). On the other hand, 11.3% (n = 110) were either jobseekers, welfare recipients, pensioners, disabled persons, interns, students, or did not provide any further information. 

The sample of the corresponding 961 frequent adolescent SM users consisted of 46.5% girls (n = 447) and 53.5% boys (n = 514). Their mean age was 13.36 years (SD = 2.36, range = 10–17). Adolescents’ (prospective) graduation level was assessed based on their current school performance. For 54.5% of adolescents (n = 524), their school performance corresponded to a higher school leaving certificate, for 35.8% (n = 344) to an intermediate school leaving certificate and for 7.2% (n = 69) to a lower school leaving certificate. However, 2.5% (n = 24) of the adolescents could not be classified regarding a graduation level, whereas 91.8% (n = 882) of the adolescents attended school, 5.9% (n = 57) were apprentices and 2.3% (n = 22) were either students, in voluntary service, serving in the military, unemployed, or engaged in activities other than those listed.

### 3.2. Factor Structure

Bartlett’s test revealed significant correlations between the nine SMDS-P items on both halves of the sample data (χ^2^(36) = 1429.87, *p* < 0.001 and χ^2^(36) = 1319.15, *p* < 0.001). The KMO criterion was 0.90 and 0.91 overall for both sub-samples and ranged between 0.88 and 0.93 for the individual items. Thus, an excellent suitability of the data for factorial analyses could be demonstrated. EFA computed on half of the sample strongly suggested a one-factor solution (eigenvalue factor 1 = 4.22; maximum very simple structure (VSS) complexity of 0.83; minimum Velicer MAP of 0.02; and minimum empirical Bayesian information criterion (BIC) of 0.02). Communalities ranged from 0.21 to 0.50. The cumulative variance explained by one factor was 0.41. The significant positive factor loadings ranged between 0.46 and 0.71 (*p* < 0.001). By applying the one-factor solution to a CFA, an excellent fit to the data was revealed (χ^2^(27) = 40.83, *p* = 0.043, ratio = 1.51; RMSEA = 0.033 (95% CI [0.006, 0.0525]); SRMR = 0.044; CFI = 0.995; TLI = 0.993). The factor loadings of all items were significantly positive (*p* < 0.001), with standardized coefficients ranging from 0.67 to 0.99. Please refer to Table 2 for EFA- and standardized CFA-factor loadings, EFA communalities, and variance proportions. Tables S2 and S3 (see Supplement) present inter-item correlations and the relative item-response frequencies.

### 3.3. Internal Consistency

A Cronbach’s α of 0.85 and a McDonald’s ω of 0.88 for the SMDS-P scale were calculated, reflecting good internal consistency.

### 3.4. Criterion Validity

**Figure 1:**
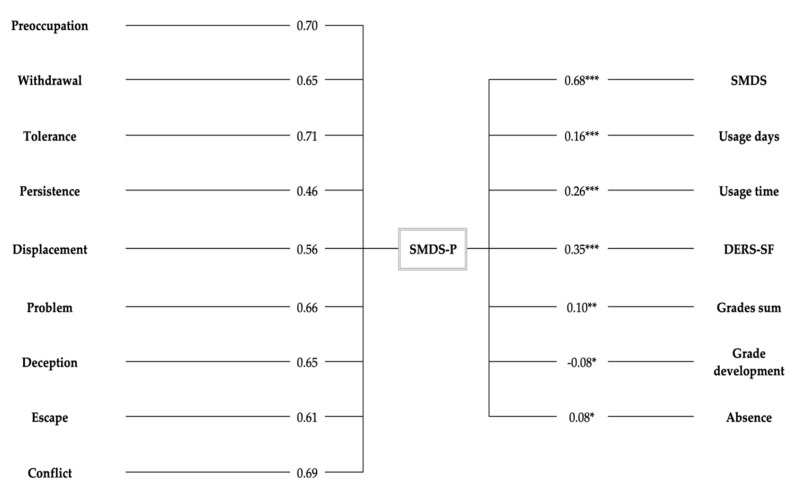
Factor loadings of the SMDS-P items on single factor and correlations with external criteria. Notes: Significant EFA SMDS-P item factor loadings are presented on the left side. Correlation coefficients with variables used to ascertain criterion validity are presented on the right side. All correlations were significant based on their *p* values with *** *p* ≤ 0.001, ** *p* ≤ 0.01, and * *p* ≤ 0.05. EFA = explanatory factor analysis, SMDS-P = Social Media Disorder Scale-Parental Version, SMDS = Social Media Disorder Scale, usage days = weekly usage frequency, usage time = average usage time per day, DERS-SF = Difficulties in Emotion Regulation Scale—Short Form, grades sum = summed grade score of the three main subjects, with higher scores indicating poorer academic performance, Absence = absence from school/work in the last three months, grades development = Change in the grade sum score in comparison with the last school report, with higher values indicating greater improvement.

Analyses revealed strong positive correlations between the total sum scores of the SMDS-P and SMDS (Pearson’s r = 0.68, *p* < 0.001). A moderate positive correlation between the sum scores of the SMDS-P and the DERS-SF (used to assess emotional dysregulation) was found (r = 0.35, *p* < 0.001). Correlations between the SMDS-*p* sum score and the average daily duration of SM usage (r = 0.26, *p* < 0.001) as well as the weekly frequency of SM use (Spearman’s ϱ = 0.16, *p* < 0.001) were weakly positive. The SMDS-P score was weakly positively correlated with the grade sum score (ϱ = 0.10, *p* = 0.006) and negatively correlated with grade development (Spearman’s ϱ = −0.08, *p* = 0.029) in a significant but negligible manner. Significant but also negligible positive correlations were computed for the SMDS-P total score with the days of absence from school or work (Spearman’s ϱ = 0.08, *p* = 0.023). All coefficients are depicted in the right column of Figure 1.

### 3.5. Sensitivity and Specificity

Adolescents were classified as problematic SM users according to their SMDS self-ratings. This classification was included into ROC curve analyses together with the SMDS-P sum score. Following Youden’s criterion, the optimal cut-off for the total SMDS-P score was 3.5 (95% CI [2.5, 4.5]) with a specificity of 80.50% (95% CI [70.69, 90.43]), a sensitivity of 87.83% (95% CI [75.65, 96.52]), and an AUC value of 90.1% (95% CI [87.4, 92.8]) indicating excellent differentiation between the problematic and uncritical users. An accuracy of 81.79% was calculated. Higher accuracy (87.30%), although at the cost of sensitivity, was obtained when applying a cut-off value of 4.5 as suggested by the DSM-5. This higher cut-off value was associated with a specificity of 89.01% and a sensitivity of 74.78%. To ensure comparability with the DSM-5 criteria and to prevent overestimation, a cut-off point of >4 was considered appropriate for the SMDS-P. 

Applying the cut-off of >4, 18.63% (95% CI [16.2, 21.1] of the adolescent SM users were classified as problematic users (N = 179). Except for sex, all dependent variables reached significance when included in an MANOVA with the cut-off based classified problematic usage (Pillai score (1, 755) = 0.69, F (10, 746) = 169.16, *p* < 0.001). For details on the post hoc MANOVA tests reported in the following, please refer to Table 3. In comparison to normal users, problematic SM users showed higher SMDS-P and SMDS values with strong effect sizes as well as higher DERS-SF sum scores and a longer daily SM usage with moderate effect sizes. Moreover, a younger age, a higher weekly frequency of SM usage, more days of school/work absence, a higher grades sum (indicating worse performance) and less academic improvement was revealed for the problematic user group compared to the normal user group with small effect sizes.

### 3.6. Accordance Rate

The classification of adolescents regarding SMD based on the SMDS-P and SMDS was associated with a kappa coefficient of 0.51. Thus, a moderate concordance between the parental and the adolescent rating was indicated.

## 4. Discussion

To the best of our knowledge, this study is the first to examine the psychometric properties of a parental questionnaire for adolescent PSMU. PSMU is a relatively new phenomenon of clinical relevance, especially in adolescents [4]. Respective screeners have, however, been constructed as self-rating scales only although young age or symptom denial might influence adolescent’s response validity [58]. The development of the Social Media Disorder Scale for Parents (SMDS-P) was based on the SMDS by van den Eijnden et al. (2016) [12]—a well-established self-rating scale for PSMU that is oriented to the diagnostic criteria for IGD. The new instrument was validated in a representative sample of 10- to 17-year-old frequent SM users and their parents (N = 961 parent–child dyads).

The presented results suggest promising psychometric properties of the SMDS-P. The internal consistency of the SMDS-P was good (Cronbach’s α = 0.85) and almost identical to the internal consistency of the SMDS (α = 0.84). The convergent validity between the SMDS-P and SMDS was strong (r = 0.68). The observed kappa coefficients indicated a moderate concordance between the ratings of parents and adolescents (kappa = 0.51). The latter is considered as satisfactory result since we assumed discrepancies between the parental assessments and self-assessments of adolescent usage patterns and considered the parental perspective as an added value for the screening. The single factor structure of the SMDS could be confirmed for the SMDS-P. The SMDS-P was able to reliably discriminate between problematic and normal adolescent SM users in terms of age, usage time including weekly frequency and daily duration, emotion regulation abilities, and academic performance including school grades and absenteeism. 

Regarding age, a small, but significant difference between the normal (mean age = 13.45, SE = 0.01) and problematic SM users (mean age = 12.95, SE = 0.16) was revealed. The slightly lower age of problematic users might indicate a greater vulnerability of younger compared to older adolescents, e.g., due to a larger imbalance between impulse control abilities and the reward system [59]. In contrast, using the SMDS, van den Eijnden et al. (2016) did not find a significant difference in age between normal and problematic SM users in samples of 10 to 17 years old [12]. However, it should be noted that the mean age of the van den Eijnden sample was 8 to 12 months higher than in the sample of the current study. An inverse relationship between age and the problematic use of SM was shown by Andreassen et al. (2018) in a sample of 16–88 year-olds [5] but no significant association could be found in the adolescent sample of van den Eijnden et al. (2018) [9]. Further research involving different age groups is desirable. 

Whereas normal users reported an average SM use of six days per week, problematic users were active on SM almost daily. The difference was small but significant. Moreover, adolescents classified with PSMU used SM significantly one hour longer (with a medium effect size) than unproblematic users. Comparatively, the results of Bányai et al. 2017 indicate that the daily usage times of normal adolescent SM users are approximately one to two hours lower than the daily usage times of adolescents with more problematic levels of SM use [60]. However, in line with the findings of van den Eijnden et al. (2016) on the SMDS [12], only weak (but significant) positive correlations were found between the SMDS-P sum score and the adolescents’ self-reported usage frequencies and duration indicating that SM time alone is not a meaningful parameter but should be seen in the context of usage patterns.

No differences between normal and problematic SM use were found in terms of gender. On the one hand, it could be assumed that SM are more likely to attract girls because they appeal to typical female psychological needs (e.g., affiliation, self-disclosure), while digital games rather tend to appeal to boys’ interests (e.g., competition, skill development) [61,62]. On the other hand, various studies reported results that do not support this assumption. Van den Eijnden et al. (2016) found more boys than girls to be engaged in PSMU only in one out of their three samples [12]. In contrast, Boer et al. (2020) reported a very weak significant positive association between female gender and problematic SM use, while Wartberg et al. (2020) and Fung (2019), in line with our results, could not find a significant gender difference at all [22,23,63]. One reason why no consistent gender effect has been revealed by now, might be the difference in SM definitions. In the present survey, YouTube was listed as an explicit example of SM based on its comment and like function. YouTube is a widely used application and is more likely to be consumed by boys than girls [64].

Notably, associations between SMDS-P and academic performance variables (school grades, grades development, and school absenteeism) were very weak to negligible in this study. A moderating influence of sex on school performance could be revealed in the longitudinal study of van den Eijnden et al. (2018): PSMU could predict worse school grades after one year for girls but not for boys [9]. Another reason for the weak association could be that PSMU is rather indirectly than directly related to academic performance and therefore only becomes apparent with a temporal delay. In the same study by van den Eijnden et al. (2018), a strong negative effect of PSMU on life satisfaction after one year was found. This effect was more pronounced in boys than in girls. According to Ng et al. (2015), life satisfaction is positively related to adolescents’ school grades [65] and could therefore act as a moderator between PSMU and academic performance, which becomes apparent later in time. Boer et al. (2020) described an overall lower school satisfaction and higher school pressure in adolescents with PSMU across all of the 29 countries investigated [23]. Moreover, Scott et al. (2019) revealed that extensive SM users were more likely to report late sleep onset and wake times on school days as well as trouble falling back asleep after night-time awakening than average users [66]. Sleep duration and quality are significant predictors of academic performance [67]. 

The clinical relevance of a scale screening for PSMU is supported by a moderate positive correlation between the SMDS-P sum score and problems in emotional regulation assessed by the DERS-SF (r = 0.35, *p* < 0.001). Furthermore, higher DERS sum scores were found in problematic compared to unproblematic SM users. Difficulties in regulating emotions have been identified as a meaningful indicator of mental health disorders such as substance and behavioral addictions including pathological gaming [68,69,70].

Our findings support the need for an instrument to predict PSMU in adolescents and could show that parents are a valid source of information. An early detection is necessary to provide appropriate treatment as fast as possible. This way, the probability of chronification and negative sequelae can be reduced. However, since Internet use has become an integral part of our everyday life, the risk of pathologizing behavior should be kept as low as possible. Therefore, the statistically proposed cut-off value of the SMDS-P has been increased to the cut-off value of the original scale to achieve higher specificity and thus reduce the rate of false positives [12,20]. 

When applying a cut-off of five fulfilled criteria, prevalence for PSMU among frequent SM users of 18.6% (95% CI [16.16, 21.09]) was estimated by the parental judgement in this study. This value seems to be higher than the estimates of van den Eijnden (7.3–11.6%) but it has to be noted that the authors included SM users irrespective of a frequent usage and did not report confidence intervals for proper comparison [12]. To reduce a potential overestimation and to be able to distinguish between at-risk and pathological users, the ICD-11 approach for problematic gaming is very promising [71]. First, in the ICD-11, pathological and at-risk gaming are separately mentioned (as Gaming Disorder (GD) and Hazardous Gaming). Second, for a GD diagnosis, in addition to usage-specific symptoms (1) impaired control over gaming, (2) increasing priority given to gaming over other activities, and (3) continuation or escalation of gaming despite the occurrence of negative consequences), clinically significant distress or the impairment of personal, social, educational, work-related, and financial functions must be fulfilled. Thus, in contrast to the monothetic DSM-5 approach, symptoms and disability criteria need to be present. An ICD-11 criteria-based self- and parental-rating scale to assess GD was developed by the authors of this study [38,39]. An underlying two-factorial structure could be revealed for both scales including cognitive–behavioral symptoms and the negative consequences of the behavior. It would be interesting to see if these results could also be applied to pathological SM usage by an adaption of GD criteria.

With the development and validation of the parental version of the Social Media Disorder Scale (SMDS-P), an additional value to the emerging research area of Internet-related disorders could be shown. The SMDS-P could support an early detection of problematic usage behavior in the especially vulnerable group of adolescents. In addition, in times of increasing concern about children’s usage behavior as digitization progresses, a parental questionnaire can not only provide an initial indication for the existence of a treatment-deserving problem but also reduce the cause for concern in the case of intense but uncritical usage behavior.

Future studies should investigate the effects of sociodemographic factors on accordance rates, such as parental or adolescent sex and the educational background, by oversampling problematic SM users. Moreover, with respect to the findings on risk factors for the PSMU of Stockdale el al. (2020) and Dalvi-Esfahani et al. (2019), classifying SM users by underlying motives (such as to socially connect and fight boredom), personality traits (such as openness to experience and loneliness), or comorbid disorders (such as depression) would be of great interest [72,73]. The clinical validation of the SMDS-P in future studies is encouraged to support its broad application in the clinical settings. Aebi et al. (2017) could show that combing self- and external ratings is of substantial diagnostic value [58] but questionnaires should not be solely relied on for clinical diagnosis. Complementary to clinical expertise, the SMDS-P might be used as a screening instrument, e.g., before appointments or when self-ratings are not available.

### Limitations

Frequent SM users and their respective parents were drawn from a representative sample. However, representativity might have been reduced since the study was conducted as an online study. Thus, households without Internet access (about 5% in Germany) [74] could not be considered. Furthermore, 94 parent–child dyads had to be excluded due to important missing data. The latter is a common disadvantage of online surveys which is outweighed by their highly economic characteristics. Moreover, using an online survey setting, we cannot guarantee that the participants were not influenced by external circumstances or the presence of other persons in their response behavior. Although, additional (objective) measures such as logged usage times had been desirable, these would have posed a particularly high juridical and technical burden (data protection) and could as well rise doubts in the anonymity of data analyses. Therefore, an increased measurement error in online studies with large samples would have been a consequence. Furthermore, it should be emphasized that this study does not include longitudinal data and therefore no statements can be made about the cause and effect of PSMU as well as the possible long-term effects. The major limitation of the current study is the lack of the clinical validation of the SMDS-P. To our knowledge, clinically validated instruments to assess PSMU in adolescents are not yet available, neither in self nor in external ratings. This would be the gold standard to allow an estimation of the clinical significance of potentially problematic behavior even though a diagnosis cannot be given at this stage.

## 5. Conclusions

Adolescents are considered particularly vulnerable to developing PSMU. Screening instruments available to date have been solely relied on self-ratings despite potential limitations due to young age or symptom denial of respondents. Thus, the SMDS-P closes a gap in the assessment of PSMU as the first successfully validated screening tool to differentiate normal and problematic adolescent SM use by parental ratings with good accuracy. Psychometric properties were comparable to the original self-rating SMDS including a one-factorial structure and an identical cut-off value. The accordance with adolescent self-ratings was moderate. The internal consistency was also good as well as the criterion validity. The scale is short and can be easily applied, making it a suitable candidate for clinical settings or research surveys. It can supplement assessments based on self-ratings or provide an initial evaluation when no self-rating is available. This enhances early PSMU detection to prevent symptom aggravation or chronification. Clinical validation in future studies is warranted.

## Figures and Tables

**Table 1:** The 9-Item Social Media Disorder Scale—Parental Version (SMDS-P) and the corresponding the Diagnostic and Statistical Manual of Mental Disorders (DSM-5) criteria.

Item	Criterion	During the Past Year, Has Your Son/Daughter…
1	Preoccupation	regularly found that he/she can’t think of anything else but the moment that he/she will be able to use social media again?
2	Withdrawal	often felt bad when he/she could not use social media?
3	Tolerance	regularly felt dissatisfied because he/she wanted to spend more time on social media?
4	Persistence	tried to spend less time on social media, but failed?
5	Displacement	regularly neglected other activities (e.g., hobbies, sport) because he/she wanted to use social media?
6	Problem	regularly had arguments with others because of his/her social media use?
7	Deception	regularly lied to you, your family, or friends about the amount of time he/she spend on social media?
8	Escape	often used social media to escape from negative feelings?
9	Conflict	had serious conflict with you, your partner, his/her brother(s) or sister(s) because of his/her social media use?

Notes: The nine criteria are based on the DSM-5 criteria for Internet Gaming Disorder. Answers can be given in a dichotomous response format with “no” (0) or “yes” (1). DSM-5 = 5th revision of the Diagnostic and Statistical Manual of Mental Disorders. Sum scores greater than four were associated with problematic social media use.

**Table 2:** Significant factor loadings, communalities, and variance proportion.

SDMS-P Item ^a^	Factor Loadings		Communalities
	EFA	CFA	
Item 1	0.70	0.87	0.48
Item 2	0.65	0.88	0.42
Item 3	0.71	0.81	0.50
Item 4	0.46	0.67	0.21
Item 5	0.56	0.72	0.32
Item 6	0.66	0.73	0.44
Item 7	0.65	0.84	0.42
Item 8	0.61	0.76	0.37
Item 9	0.69	0.83	0.48
**Variance Proportion ^b^**	0.41		

Notes: SMDS-P = Social Media Disorder Scale-Parental Version, EFA = exploratory factor analysis, CFA = confirmatory factor analysis. ^a^ Item descriptions are presented in Table 1; and ^b^ proportion of explained variance by single EFA factor.

**Table 3:** Post hoc MANOVA and between the group tests of SM usage groups.

	SM Usage		Post Hoc Tests	
Variables	Normal	Problematic	(χ^2^/Scheffé)	Cohen’s d/Cramer’s V
Absolute frequency	782	179	-	-
Relative frequency in % (95% CI)	81.37 [83.84; 78.8]	18.63 [16.16; 21.09]	-	-
Age mean (SE)	13.45 (0.01)	12.95 (0.16)	−0.5 **	0.21
Female sex in % (95% CI)	47.95 [44.45; 51.64]	40.22 [33.04; 47.41]	-	-
SMDS-P sum score mean (SE)	1.13 (0.05)	6.77 (0.11)	5.64 ***	4.02
SMDS sum score mean (SE)	1.04 (0.06)	4.31 (0.21)	3.27 ***	1.74
Days of social media use per week mean (SE)	6.01 (0.07)	6.55 (0.11)	0.54 ***	0.3
Minutes of social media use per day mean (SE)	144.64 (8.6)	207.33 (11.43)	62.69 ***	0.51
DERS sum score mean (SE)	38.76 (0.42)	46.98 (0.96)	8.22 ***	0.69
Days of absence mean (SE)	1.67 (0.13)	2.9 (0.44)	1.23 ***	0.3
Grades sum mean (SE)	6.18 (0.09)	6.85 (0.2)	0.67 **	0.27
Grades development mean (SE)	3.24 (0.02)	3.08 (0.05)	−0.16 **	0.24

Notes: *p*-values: *** *p* ≤ 0.001, ** *p* ≤ 0.01, MANOVA = multivariate analysis of variance, χ^2^ = chi-square, Cohen’s d = effect sizes, (95% CI) = 95% confidence interval, SE = standard error of the mean, SMDS-P = Social Media Disorder Scale-Parental Version, SMDS = Social Media Disorder Scale, DERS-SF = Difficulties in Emotion Regulation Scale – Short Form, days of absence in school/apprenticeship/job, grades sum = cumulated grades of the three main subjects with higher scores indicating poorer performance, grades development = improvement of grades sum over last term.

## Data Availability

The data that support the findings of this study are available from the corresponding author (KP) upon reasonable request after all results of the parent-child survey have been published.

## Supplementary Materials

The following are available online at https://www.mdpi.com/2077-0383/10/4/617/s1, Table S1: SMDS-P German version; Table S2: Inter-item correlation SMDS-P; Table S3: Relative item response frequency of the SMDS-P.
