# Supplementary material for: Assessing Problematic Social Media Use in Adolescents by Parental Ratings: Development and Validation of the Social Media Disorder Scale for Parents (SMDS-P)
Source: J Clin Med. 2021 Feb 6;10(4):617. doi: 10.3390/jcm10040617 (PMC7914646; doi:10.3390/jcm10040617)
Supplement: Supplementary file 1 [file jcm-10-00617-s001.pdf]

Table S1. SMDS-P German Version.

	**Hat Ihr Kind im vergangenen Jahr...**		
**1.**	schon mal stundenlang an nichts anderes denken können, als an den Moment an dem er/sie wieder soziale Medien nutzen kann?	☐Nein	☐ Ja
**2.**	sich unzufrieden gefühlt, weil er/sie soziale Medien häufiger nutzen wollte?	☐Nein	☐ Ja
**3.**	sich unglücklich gefühlt, wenn er/sie soziale Medien nicht nutzen konnte?	☐Nein	☐ Ja
**4.**	die Nutzung sozialer Medien nicht verringern können, während andere ihm/ihr sagten, dass er/sie das dringend tun muss?	☐Nein	☐ Ja
**5.**	soziale Medien genutzt, um nicht an unangenehme Dinge denken zu müssen?	☐Nein	☐ Ja
**6.**	Streit mit anderen gehabt, durch sein/ihr Nutzungsverhalten?	☐Nein	☐ Ja
**7.**	die Zeit, die er/sie sozialen Medien gewidmet hat, vor anderen geheim gehalten?	☐Nein	☐ Ja
**8.**	kein Interesse an Hobbys oder anderen Aktivitäten gezeigt, weil er/sie lieber soziale Medien nutzen wollte?	☐Nein	☐ Ja
**9.**	ernsthafte Probleme mit der Familie, Freunden oder dem Partner durch die Nutzung sozialer Medien gehabt?	☐Nein	☐ Ja

Notes: SMDS-P = Social Media Disorder Scale - Parental Version in original German language.

Table S2. Inter-item correlation SMDS-P.[a]

	**Item 1**	**Item 2**	**Item 3**	**Item 4**	**Item 5**	**Item 6**	**Item 7**	**Item 8**	**Item 9**
**Item 1**	1.00								
**Item 2**	0.55	1.00							
**Item 3**	0.54	0.57	1.00						
**Item 4**	0.39	0.26	0.26	1.00					
**Item 5**	0.33	0.34	0.34	0.29	1.00				
**Item 6**	0.44	0.41	0.40	0.30	0.35	1.00			
**Item 7**	0.43	0.40	0.40	0.33	0.41	0.43	1.00		
**Item 8**	0.39	0.41	0.41	0.29	0.38	0.40	0.38	1.00	
**Item 9**	0.43	0.41	0.43	0.36	0.43	0.39	0.49	0.41	1.00

Notes: SMDS-P = Social Media Disorder Scale – Parental Version, [a] Item descriptions are presented in Table 1.

Table S3. Relative item-response frequency of the SMDS-P.

SMDS-P Item[a]	Response
	**Yes**
**Item 1**	0.26
**Item 2**	0.25
**Item 3**	0.27
**Item 4**	0.19
**Item 5**	0.30
**Item 6**	0.26
**Item 7**	0.17
**Item 8**	0.29
**Item 9**	0.18

Notes: SMDS-P = Social Media Disorder Scale – Parental Version, [a] Item descriptions are presented in Table 1.
